# Significant Differences in the Development of Acquired Resistance to the MDM2 Inhibitor SAR405838 between *In Vitro* and *In Vivo* Drug Treatment

**DOI:** 10.1371/journal.pone.0128807

**Published:** 2015-06-12

**Authors:** C. Gianna Hoffman-Luca, Chao-Yie Yang, Jianfeng Lu, Daniel Ziazadeh, Donna McEachern, Laurent Debussche, Shaomeng Wang

**Affiliations:** 1 University of Michigan Comprehensive Cancer Center, Ann Arbor, Michigan, United States of America; 2 Department of Pharmacology, University of Michigan, Ann Arbor, Michigan, United States of America; 3 Department of Internal Medicine, University of Michigan, Ann Arbor, Michigan, United States of America; 4 Department of Medicinal Chemistry, University of Michigan, Ann Arbor, Michigan, United States of America; 5 Sanofi Oncology, Vitry-sue-Seine, France; University of Illinois at Chicago, UNITED STATES

## Abstract

SAR405838 is a potent and specific MDM2 inhibitor currently being evaluated in Phase I clinical trials for the treatment of human cancer. Using the SJSA-1 osteosarcoma cell line which harbors an amplified *MDM2* gene and wild-type p53, we have investigated the acquired resistance mechanisms both *in vitro* and *in vivo* to SAR405838. Treatment of SJSA-1 cells with SAR405838 *in vitro* leads to dose-dependent cell growth inhibition, cell cycle arrest and robust apoptosis. However, prolonged treatment of SJSA-1 cells *in vitro* with SAR405838 results in profound acquired resistance to the drug. Analysis of *in vitro*-derived resistant cell lines showed that p53 is mutated in the DNA binding domain and can no longer be activated by SAR405838. Treatment of the parental SJSA-1 xenograft tumors with SAR405838 in mice yields rapid tumor regression but the tumors eventually regrow. Culturing the regrown tumors established a number of sublines, which showed only modest (3–5 times) loss of sensitivity to SAR405838 *in vitro*. Sequencing of the p53 showed that it retains its wild-type status in these *in vivo* sublines, with the exception of one subline, which harbors a single heterozygous C176F p53 mutation. Using xenograft models of two *in vivo* derived sublines, which has either wild-type p53 or p53 containing a single heterozygous C176F mutation, we showed that while SAR405838 effectively achieves partial tumor regression in these models, it no longer induces complete tumor regression and tumors resume growth once the treatment is stopped. Harvesting and culturing tumors obtained from a prolonged treatment with SAR405838 in mice established additional *in vivo* sublines, which all contain a single heterozygous C176F mutation with no additional p53 mutation detected. Interestingly, SAR405838 can still effectively activate p53 in all sublines containing a single heterozygous C176F mutation, with a moderately reduced potency as compared to that in the parental cell line. Consistently, SAR405838 is 3–5 times less effective in all the *in vivo* derived sublines containing a single heterozygous C176F p53 mutation than in the SJSA-1 parental cell line in assays of cell growth and apoptosis. Computational modeling suggested that a p53 tetramer containing two wild-type p53 molecules and two C176F mutated molecules can maintain the structural stability and interactions with DNA by formation of additional hydrophobic and cation-π interactions which compensate for the loss of sulphur-zinc coordination. Our data thus show that SJSA-1 tumor cells acquire very different levels of resistance *in vitro* and *in vivo* to the MDM2 inhibitor SAR405838. Our present study may have a significant implication for the investigation of resistant mechanisms for other classes of anticancer drugs.

## Introduction

The tumor suppressor protein p53 is a transcriptional factor which regulates a variety of cellular processes including, but not limited to cell cycle, apoptosis, DNA repair and senescence[1–4]. The gene encoding the p53 protein, *TP53*, is mutated in approximately 50% of human cancers, resulting in inactivation of p53 as a transcriptional factor and a loss of its tumor suppressor function[5,6]. In the remaining 50% of human cancers, the tumor suppressor function of p53 is inhibited by a number of mechanisms[7]. The oncogenic MDM2 protein is a major cellular inhibitor of wild-type p53[8–10]. In cells with wild-type p53, upon activation p53 transcribes MDM2, leading to increases in levels of both mRNA and MDM2 protein. The MDM2 protein in turn, binds to and inhibits p53 through several distinct mechanisms[11]. First, MDM2 ubiquitinates p53 and promotes p53 degradation. Second, MDM2 binds to the DNA binding domain in p53 and blocks its transcriptional activity[12]. Third, MDM2 is involved in the nuclear export of p53, rendering p53 inaccessible to its target DNAs[8,12]. Through these multiple mechanisms, MDM2 functions as an effective cellular antagonist of wild-type p53.

Because inhibition of p53 by MDM2 stems from their direct physical interaction, small-molecule inhibitors designed to bind to MDM2 and block the MDM2-p53 protein-protein interaction (hereafter called MDM2 inhibitors), can lead to accumulation and activation of wild-type p53, thus releasing the tumor suppressor function of p53 [13–17]. Recent years have seen intense research efforts aimed at the design and development of MDM2 inhibitors as a new class of therapeutic agents for the treatment of human cancer. To date, several such inhibitors[18], including SAR405838 (also known as MI-77301) designed in our laboratory[19], have progressed into clinical development.

For successful clinical development of potent and specific MDM2 inhibitors as a new class of anticancer drugs, an understanding of both *de novo* and acquired resistance mechanisms is critical to select patients whose tumors are most likely to respond to the treatment and to develop rational strategies to overcome the resistance. Since potent and specific MDM2 inhibitors activate only wild-type p53, their cellular activity is restricted to tumor cells with wild-type p53, suggesting the possibility that tumor cells can develop acquired resistance to MDM2 inhibitors by inactivating p53 [20–22]. Indeed, previous investigations have demonstrated that when cancer cell lines with wild-type p53 status are treated *in vitro* for a prolonged period with nutlin-3, a potent and specific MDM2 inhibitor, tumor cells acquire inactivating p53 mutation(s), which renders p53 non-functional and results in profound acquired resistance to the drug[23–26]. We have recently shown that when acute leukemia RS4;11 and MV4;11 cell lines are treated with SAR405838 either *in vitro* or *in vivo*, they also became highly resistant to SAR405838, as a result of p53 mutation(s)[27].

In the present study, we investigated the resistance mechanisms for our MDM2 inhibitor SAR405838 in the SJSA-1 osteosarcoma cell line *in vitro* and *in vivo*. The SJSA-1 osteosarcoma cell line contains an amplified MDM2 gene and wild-type p53. Our previous study showed that SAR405838 effectively induces apoptosis in the SJSA-1 osteosarcoma cell line *in vitro* and in the xenograft tumor tissue *in vivo*[19]. Furthermore, SAR405838 yields rapid, complete and persistent tumor regression in the SJSA-1 xenograft model in mice,[19] and consequently, this osteosarcoma cell line is an excellent model with which to investigate the resistance mechanisms of SAR405838.

Our data clearly show that the resistance acquired when the SJSA-1 cell line is treated in cell culture is very different from that when the SJSA-1 xenograft tumors are treated in animals. Our present study has yielded new insights into the *in vitro* and *in vivo* resistance mechanisms of SAR405838.

## Materials and Methods

### Reagents and antibodies

SAR405838 was synthesized using a method similar to that used for MI-888 [28]. The following primary antibodies were used: MDM2 (SMP-14, sc-965) and GAPDH (sc-5778) from Santa Cruz Biotechnology, p53 (DO-1, OP43) from Millipore and p21 (12D1) from Cell Signaling.

### Cell culture, cell viability, and apoptosis assays

SJSA-1 cell lines were purchased from American Type Culture Collection (ATCC) and cultured as recommended. Cell viability was evaluated by a WST-8 assay[29]. Apoptosis was analyzed using Annexin V-FLUOS staining kit (Roche Applied Science, Indianapolis, IN).

### Stable short hairpin interfering RNA constructs

A previous study was used to guide the generation of short 19-bp hairpins for RNA interference: p53 (nucleotides 611–629 Genbank NM000546)[30]. The oligonucleotides were annealed and ligated into a self-inactivating lentiviral vector under the control of the H1 promoter[31]. The vector used was also designed to carry the GFP reporter gene under control of the human ubiquitin-C promoter in order to monitor infection efficiency. A scrambled shRNA construct was also utilized as a control[30]. Lentiviral shRNA virus-containing supernatant was generated by the University of Michigan Vector Core. Virus-containing supernatant was used to infect SJSA-1 cells. The cells were sorted 96 h post-infection for GFP fluorescence and used for all subsequent experiments.

### Analysis of p53 mutation

Mutation of p53 was determined by sequence analysis. Total RNA was extracted with RNeasy Mini Kit from Qiagen Inc (Valencia, CA). Complementary DNA (cDNA) was prepared by reverse transcription using SuperScript III First-Strand Synthesis SyperMix system from Invitrogen Corporation (Carlsbad, CA) following the manufacturer's protocol. Primers to amplify and sequence cDNA for exons 2 to 11 of human p53 were used from Aziz *et al*. [23]. The amplified p53 cDNA was sequenced by the University of Michigan Sequencing Core. Mutation Surveyor (SoftGenetics LLC) software was used to compare experimental sequences against Refseq GenBank as well as by visual inspection of sequence tracings.

### *In vivo* Xenograft studies in mice

To develop xenograft tumors, 5 x 10^6^ tumor cells with 50% Matrigel were injected subcutaneously on the dorsal side of SCID mice. Each treatment group consisted of 6–8 mice. Tumor sizes and animal weights were measured 2–3 times per week with tumor volume (mm^3^) = (length x width^2^)/2. Tumor growth inhibition was calculated by the formula: 100% x (mean volume of controls—mean volume of treated)/mean volume of controls at treatment end. All the animal experiments performed in this study have been approved by the University of Michigan Committee for Use and Care of Animals.

### Statistical analysis

Differences in mean values of cell apoptosis among different groups were analyzed by 2-way ANOVA. For *in vivo* studies, significance (*P*) was calculated by Students *t* test, with a *P* value <0.05 being considered significant. All statistical tests were 2-sided, and all statistical analysis was carried out using GraphPad Prism 6.

### Computational modeling

The crystal structure of tetrameric p53 and the DNA of the BAX response element (PDB entry: 4HEJ) [32] was used in the computational modeling. For the molecular dynamics (MD) simulations, we used the MOE program [31] to determine the protonation state of ionizable groups on p53 under standard physiological conditions. The MOE program was also used to perform the mutation of C176F in p53. In modeling of C176F, we selected the Phe rotamer that has no van der Waal clashes with neighboring atoms. PMEMD from Amber (version 12) [33] was used for MD simulations. The Amber 99SB force field parameters [34] were used for the amino acids. The force field parameters for the zinc ion were taken from Lu et. al [35].

To prepare the topology and coordinate files, counter ions were first added to neutralize the charges in p53/DNA and the system was then placed in a 13Å octahedral box of water (the TIP3P [36] water model). A minimization procedure of 1–1000 steps using conjugated gradient was carried out first and was followed by 2000 steps of steepest descent. Then a 500 ps constant volume and constant temperature (NVT) simulation was performed to raise the temperature of the system to 298K while constraining backbone atoms with a 5 kcal/mol/Å^2^ force constant with reference to the crystal structure. A second 200 ps constant pressure and constant temperature (NPT) simulation at 298K was performed while constraining backbone atoms with a 2 kcal/mol/Å^2^ force constant with reference to the crystal structure. The system was then subjected to the 6 ns production run in the isobaric isothermal (NPT, T = 298K and P = 1 atm) ensemble. A harmonic constraint with a 2 kcal/mol/Å^2^ force constant was applied to the backbone atoms of the DNA double helix to ensure its integrity during the simulations. The SHAKE [37] algorithm was used to fix bonds involving hydrogen during the simulation. The PME method [38] was used and the non-bonded cutoff distance was set at 12Å. The time step was 2 fs, and neighboring pairs list was updated every 20 steps. All graphics were prepared using the PyMol program.

## Results

### Establishment and characterization of *in vitro* SJSA-1 sublines resistant to SAR405838

**Fig 1:**
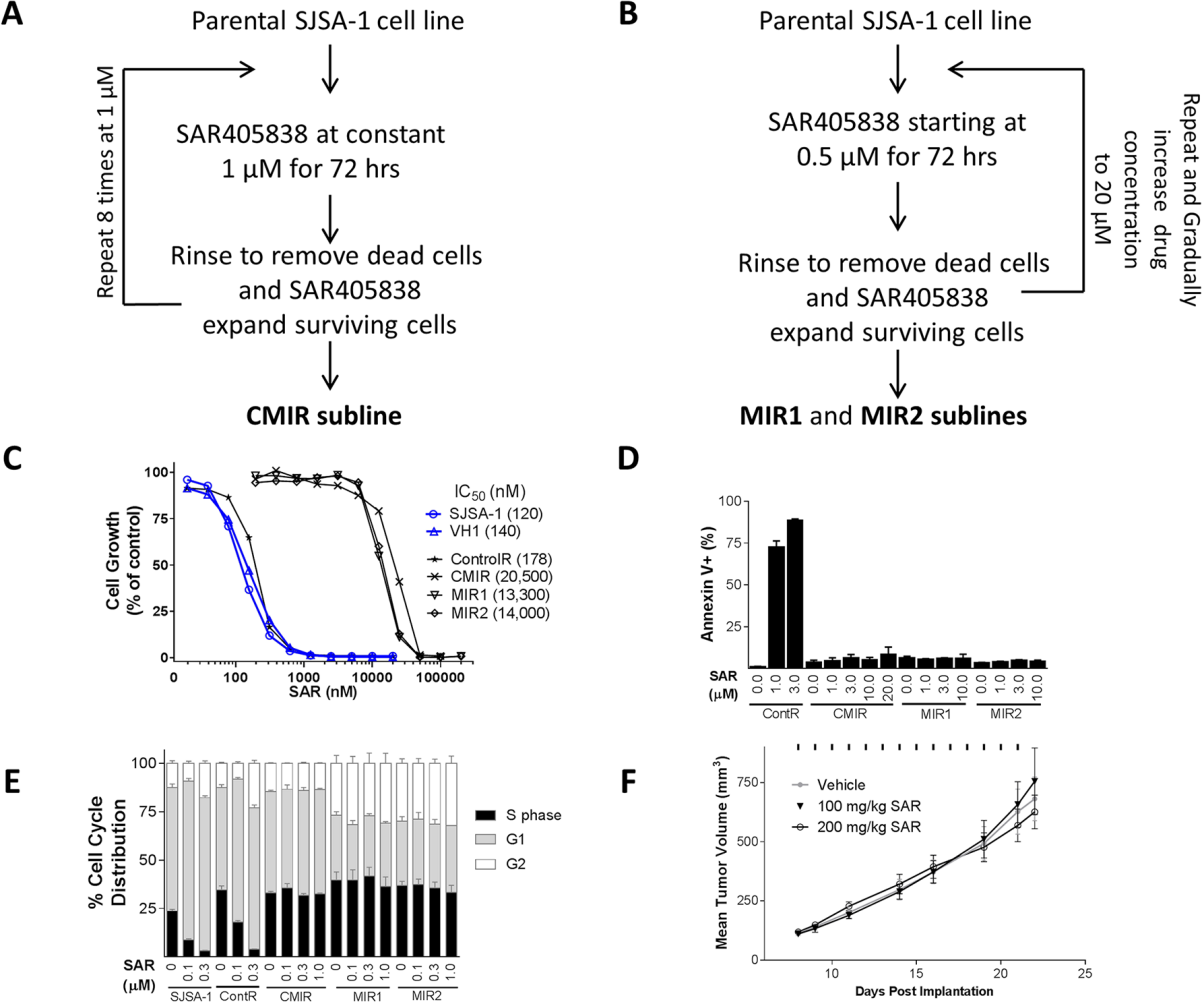
Establishment and characterization of *in vitro* SJSA-1 resistant sublines. A, Protocol to obtain the CMIR resistant cell line by treating the SJSA-1 cells with a constant concentration of SAR405838. B, Protocol to establish the MIR1 and MIR2 cell lines by treating the SJSA-1 cells with stepwise increments of concentration of SAR405838. C, Cell growth inhibition of SAR405838 in SJSA-1 parental, three resistant and two control cell lines. Cell lines were treated with SAR405838 for 4 days and cell viability was determined by a WST assay. D, Induction of apoptosis by SAR405838 in three resistant and one control cell line. Cells were treated with SAR405838 for 48 h and stained with Annexin V/PI for flow cytometry. Data (mean ± SD) are from triplicates, including both early (Annexin V-positive/PI-negative) and late (Annexin V-positive/PI-positive) apoptotic cells. E, Cell cycle effect of SAR405838 in three resistant cell lines, one control cell line and the parental SJSA-1 cell line. Cells were treated with SAR405838 for 24 h for cell cycle analysis by flow cytometry with P.I. staining. F, Antitumor activity of SAR405838 on xenograft tumors established using the MIR2 cell line in mice. SCID mice bearing MIR2 xenograft tumors were treated with vehicle, 100 mg/kg or 200 mg/kg of SAR405838 by oral gavage for 14 days.

To develop acquired resistance of the SJSA-1 cell line to SAR405838 *in vitro*, the SJSA-1 cells were treated with SAR405838 in cell culture using two different protocols (Fig 1), similar to those used previously in generation of resistant cell lines to adriamycin[39]. In the first protocol (Fig 1A), the SJSA-1 parental cell line was treated with 1 μM of SAR405838 for 72 h. The cells were then thoroughly washed to remove dead cells and SAR405838. The surviving cells were expanded in regular cell culture medium and treated again with 1 μM of SAR405838 for 72 hr. Repeating the process 8 times yielded the CMIR subline. In the second protocol (Fig 1B), the SJSA-1 parental cell line was treated with SAR405838 in stepwise increments of concentration, starting from 0.5 μM and eventually increased to 20 μM. This yielded two sublines (MIR1 and MIR2). We also obtained a control cell line by treating the parental SJSA-1 cell line with the vehicle.

In a cell growth assay, these three *in vitro-*derived SJSA-1 sublines (CMIR, MIR1 and MIR2) all exhibit >100-fold resistance to SAR405838, based upon the IC_50_ values, when compared to the parental cell line and the vehicle-treated control cell line (Fig 1C). Furthermore, while SAR405838 is very effective in inducing apoptosis and cell cycle arrest in either the control or the parental cell line, it has no effect in these three *in vitro-*derived SJSA-1 sublines (Fig 1D and 1E).

Since the activity of MDM2 inhibitors is known to depend upon wild-type p53, we analyzed p53 mutation by sequencing exons 2–11 in each of the three *in vitro* resistant sublines, (CMIR, MIR1 and MIR2) and found that they all harbor p53 mutation(s) in the DNA binding domain (S1A Table).

**Fig 2:**
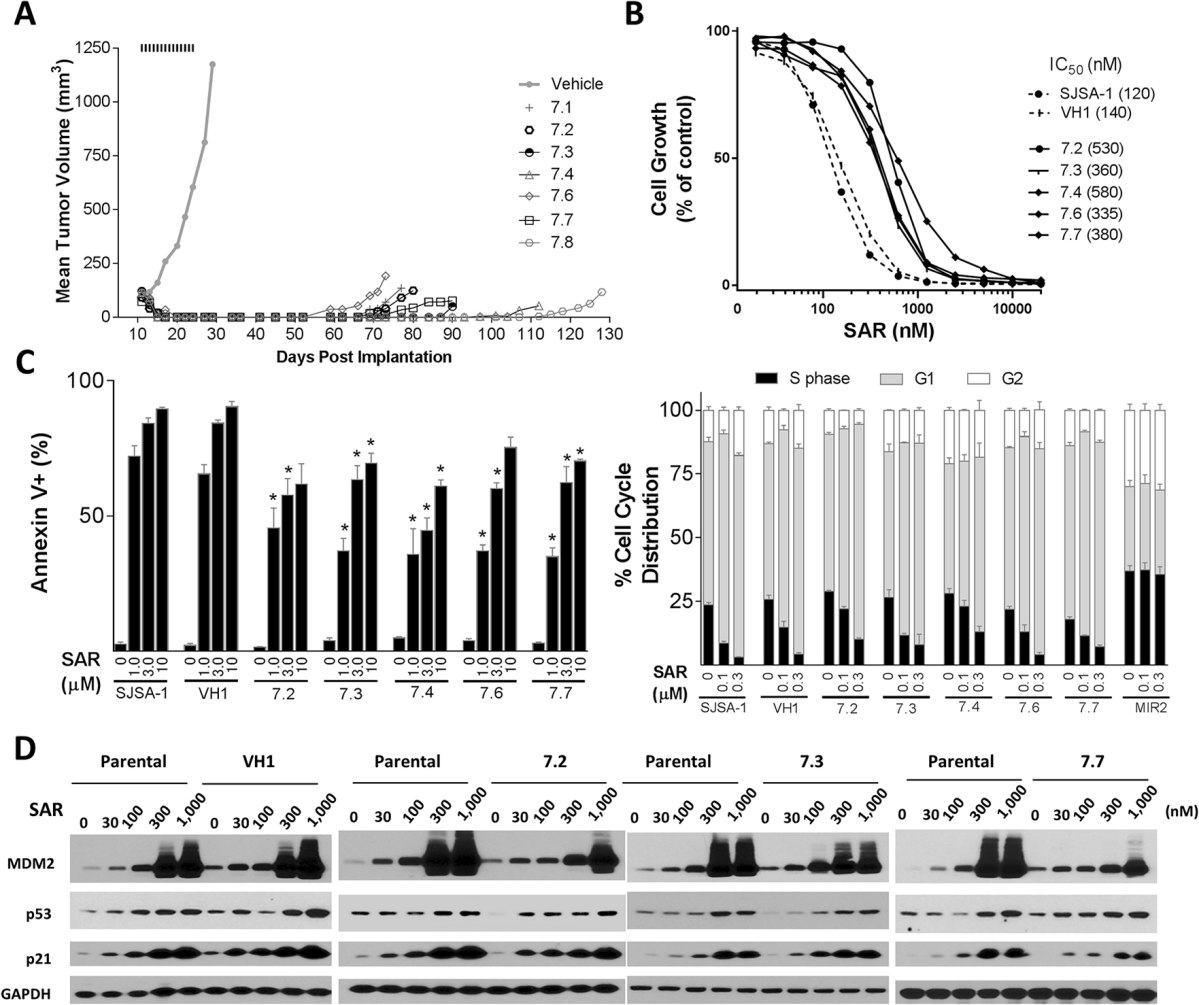
Establishment and characterization of *in vivo* sublines established from SJSA-1 tumors treated with SAR405838 in mice. A, SJSA-1 xenograft tumors were treated with vehicle or 100 mg/kg of SAR405838 orally for 14 days. When regressed SJSA-1 tumors treated with SAR405838 regrew, they were harvested and cultured to establish several *in vivo* sublines. One control subline was established from a tumor treated with vehicle. B, Inhibition of cell growth by SAR405838 in several *in vivo* SJSA-1 sublines (7.2–7.4, 7.6 and 7.7), one vehicle control subline (VH1) and the parental SJSA-1 cell line. Cells were treated with SAR405838 for 4 days and cell viability was determined by a WST assay. C, Examination of apoptosis induction and cell cycle arrest by SAR405838 in several *in vivo* SJSA-1 sublines, one vehicle control subline (VH1) and the parental SJSA-1 cell line. Cells were treated with SAR405838 for 48 h for apoptosis analysis by flow cytometry with Annexin V/PI double staining. The test was used to compare *in vivo* sublines to parental SJSA-1 cells. *, *P* < 0.05. Cells were treated with SAR405838 for 24 h for cell cycle analysis by flow cytometry with P.I. staining. D, Western blot analysis of p53, MDM2, p21 and GAPDH protein level in SJSA-1 parental, vehicle (VH1) and *in vivo* sublines (7.2, 7.3 & 7.7). Cells were treated with SAR405838 for 24 h at indicated concentrations.

To further assess the resistance of these *in vitro-*derived sublines, we investigated the *in vivo* efficacy of SAR405838 in xenograft tumors in mice, established using the MIR2 subline containing the heterozygous R273C mutation. Oral administration of SAR405838 at both 100 mg/kg and 200 mg/kg daily for two weeks failed to show any antitumor activity against MIR2 xenograft tumors, as compared to the vehicle treated control (Fig 1F and S1A Fig). In comparison, SAR405838 at 100 mg/kg daily dosing induces rapid and complete regression of the SJSA-1 xenograft tumors established using the SJSA-1 parental cell line (Fig 2A).

We generated several subline lines by harvesting and culturing the MIR2 tumors treated with 200 mg/kg of SAR405838 and found that each of them harbors the same heterozygous R273C mutation as in the MIR2 subline (S1B Table).

These data show that treatment of the SJSA-1 cell line *in vitro* with SAR405838 yielded sublines with p53 mutations, R273C in two sublines and L130R and C277F in the third subline. All these mutations are located in the DNA-binding domain. Furthermore, these sublines are highly resistant to SAR405838 in both *in vitro* and *in vivo* assays.

### SJSA-1 sublines treated *in vivo* with a single round of SAR405838

Our previous study[19] showed that SAR405838 induced rapid regression of the SJSA-1 xenograft tumors, but all tumors eventually regrew after the treatment was stopped, suggesting acquired resistance *in vivo*. We investigated possible *in vivo* acquired resistance mechanisms of the SJSA-1 tumors in mice when treated with SAR405838.

Consistent with our previous data[19], oral administration of SAR405838 at 100 mg/kg, daily for 2 weeks induces rapid and complete tumor regression of the SJSA-1 xenograft tumors and while the tumor regression persists for >40 days, all tumors eventually regrow (Fig 2A and S1B Fig). We isolated SJSA-1 tumor cells from vehicle-treated tumors and SAR405838-treated regrown tumors and established several sublines. The vehicle treated subline (VH1) is equally sensitive to SAR405838 when compared to the parental cell line in a cell growth assay. Surprisingly, in contrast to the profound acquired resistance observed for those three sublines obtained from *in vitro* treatment of the SJSA-1 cells with SAR405838, the five sublines (sublines 7.2–7.4 and 7.6–7.7) obtained from SAR405838-treated, regrown SJSA-1 xenograft tumors exhibit only a modest change in their sensitivity to SAR405838, with IC_50_ values in a cell growth assay 3–5 times higher than in the parental SJSA-1 cell line (Fig 2B). Apoptosis and cell cycle analysis by flow cytometry further showed that these *in vivo* sublines indeed remain responsive to SAR405838, albeit with modestly reduced sensitivity (Fig 2C).

We next analyzed the p53 mutation status of these five *in vivo* sublines and found that four out of them retain their wild-type p53 status. The exception was the 7.2 subline, which harbors a single heterozygous mutation C176F (S2 Table).

To examine if p53 is still functional in these sublines, we examined induction of p21 and MDM2 and accumulation of p53 protein by SAR405838. In three representative sublines, including the 7.2 subline, SAR405838 induces dose-dependent increases of MDM2, p21 and p53 proteins, indicating that p53 is still functional (Fig 2D).

These data clearly show that these *in vivo* sublines established from the regressed, regrown SJSA-1 tumors treated with SAR405838 contain functional p53, even when the subline contains a heterozygous C176F mutation, and are only marginally less sensitive to SAR405838 than the parental cell line.

### Further treatment of 7.6 SJSA-1 subline xenograft tumors with SAR405838 in mice

Cancer patients are often treated with multiple rounds of a therapy until disease progression occurs and accordingly, we next investigated if a more profound resistance to SAR405838 can be developed when the xenograft tumors established from the 7.6 SJSA-1 subline containing a wild-type p53 are treated with SAR405838 in mice.

**Fig 3:**
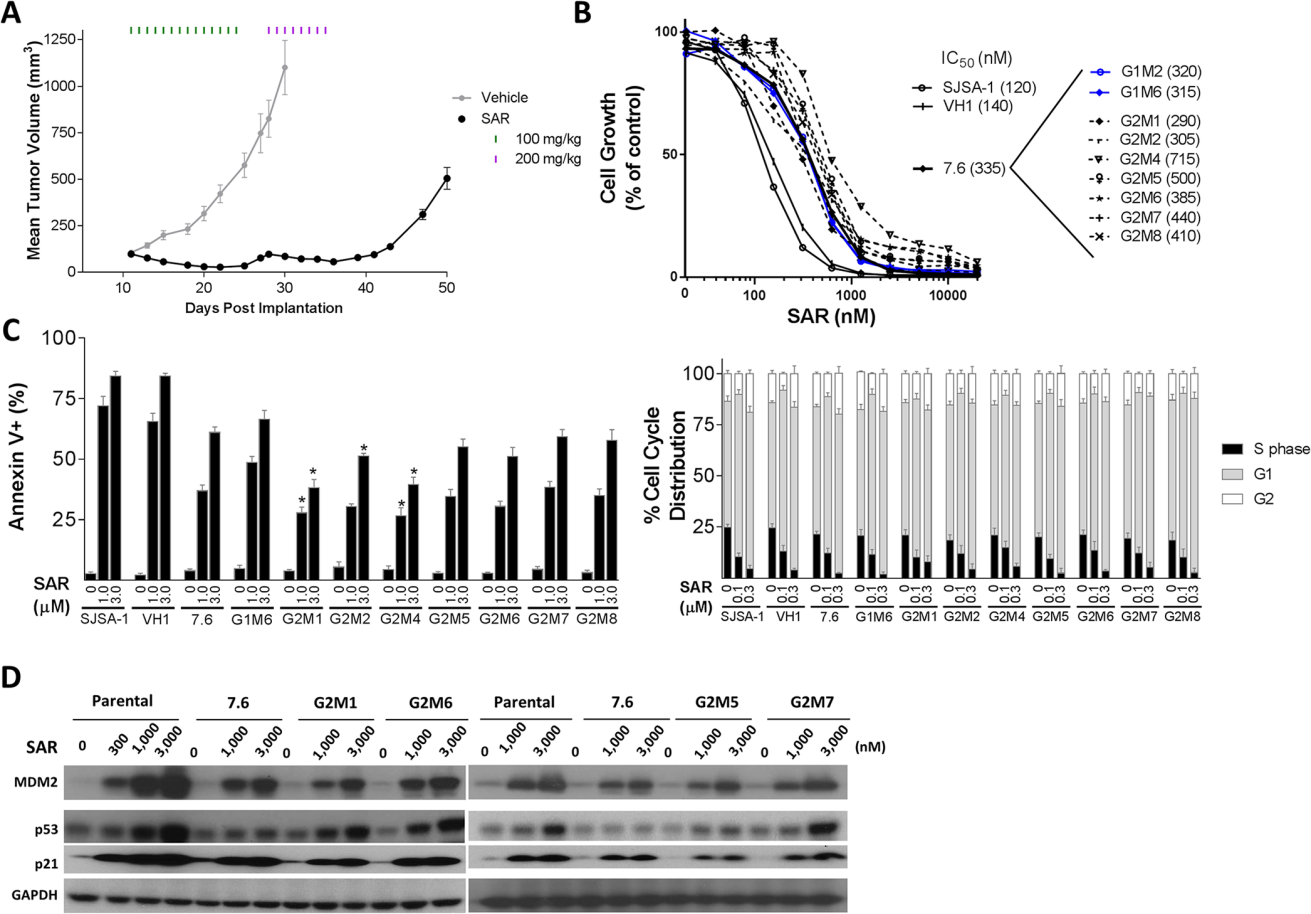
Establishment and characterization of sublines from treatment of 7.6 SJSA-1xenograft tumors with SAR405838 in mice. A, 7.6 SJSA-1 xenograft tumors were treated with vehicle or 100 mg/kg of SAR405838 orally for 14 days, followed by 200 mg/kg SAR405838 for 8 days. When tumors treated with SAR405838 grew to 500 mm^3^, they were harvested and cultured to establish 7 sublines (G2M1-G2M2, G2M4-G2M8). Two sublines (G1M2 and G1M6) were also established by harvesting and culturing vehicle-treated 7.6 SJSA-1 tumors. B, Cell growth inhibition of SAR405838 in sublines obtained from 7.6 SJSA-1 tumors, the SJSA-1 parental cell line and a subline (VH1) established from a vehicle-treated SJSA-1 tumor from the experiment shown in Fig 2. Cells were treated with SAR405838 for 4 days and cell viability was evaluated by a WST assay. C, Analysis of apoptosis and cell cycle in G2M1-G2M2, G2M4-G2M8 sublines, the SJSA-1 parental cell line and VH1 cell line. Cells were treated with SAR405838 for 48 h for apoptosis analysis by flow cytometry with Annexin V/P.I. double staining. The test was used to compare sublines (G2M1, G2M2, G2M4-G2M8) to 7.6 subline. *, *P* < 0.05. Cells were treated with SAR405838 for 24 h for cell cycle analysis by flow cytometry with P.I. staining. D, Western blot analysis of p53, MDM2, p21 and GAPDH protein level in G2M1, G2M6, G2M5 and G2M7 sublines, SJSA-1 parental cell line and 7.6 subline. Cells were treated for 24 h with SAR405838 at indicated concentrations of SAR405838 and individual protein was probed with a specific antibody.

When the 7.6 SJSA-1 xenograft tumors reached approximately 100 mm^3^ in volume, they were treated with SAR405838 at 100 mg/kg, daily for 14 days (Fig 3A). This treatment yielded 72% tumor regression on average during the treatment period. After the treatment was stopped, tumors resumed growth and reached 100 mm^3^ in size on average within three days (day 28). Since SAR405838 at 100 mg/kg did not yield complete tumor regression, we next treated the tumors with SAR405838 at 200 mg/kg, daily for 8 days, which resulted in tumor regression of 40% on average during the treatment with no sign of toxicity (Fig 3A and S1C Fig). When the SAR405838 treatment was stopped, the tumors resumed growth and were then harvested and cultured to generate seven sublines (G2M1, G2M2, G2M4—G2M8) along with two representative sublines (G1M2 and G1M6) established from the vehicle treated tumors (Fig 3).

Cell growth assay showed that these seven sublines (G2M1, G2M2, G2M4-G2M8) are 3–5 times less sensitive to SAR405838 than the parental cell line and are essentially equally sensitive to SAR405838 as the two sublines (G1M2 and G1M6) established from two 7.6 tumors treated with the vehicle. Flow cytometric analysis further showed that SAR405838 is still quite effective in induction of apoptosis in all 7 sublines (G2M1, G2M2, G2M4—G2M8), with moderately reduced potency, when compared to the SJSA-1 parental cell line and a control cell line (VH1) (Fig 3C). Furthermore, SAR405838 is equally effective and potent in induction of cell cycle arrest in all of the sublines obtained from prolonged treatment of SAR405838 *in vivo*, when compared to all the control cell lines, including the SJSA-1 parental cell line (Fig 3C).

Analysis of the p53 mutation status of these 7 sublines (G2M1, G2M2, and G2M4—G2M8) showed that they all harbor a single heterozygous C176F mutation (S3 Table), and in order to assess if these sublines still contain functional p53, we examined induction of p21, MDM2 and p53 proteins by SAR405838. Western blot analysis showed that SAR405838 clearly induces a dose-dependent increase of MDM2, p21 and p53 proteins (Fig 3D) in 4 representative sublines, indicating that p53 remains functional in these sublines.

Hence, our data showed that prolonged treatment in mice with SAR405838 of the xenograft tumors established using the 7.6 subline containing wild-type 53 uniformly yielded a single heterozygous C176F p53 mutation in all 7 sublines but p53 remains functionally active. Consistently, SAR405838 is still effective in these 7 sublines in inhibition of cell growth and induction of apoptosis and is only 3–5 less potent than in the parental SJSA-1 cell line.

### Further treatment of xenograft tumors of the 7.2 SJSA-1 subline with SAR405838 in mice

The SJSA-1 7.2 subline, established from a SJSA-1 tumor treated daily for two weeks with 100 mg/kg of SAR405838, contains a single heterozygous C176F mutation of its p53 but is only moderately less sensitive to SAR405838 than the parental SJSA-1 cell line in several assays. We investigated whether the SJSA-1 7.2 subline can develop increased resistance to SAR405838 with further SAR405838 treatment *in vivo*.

**Fig 4:**
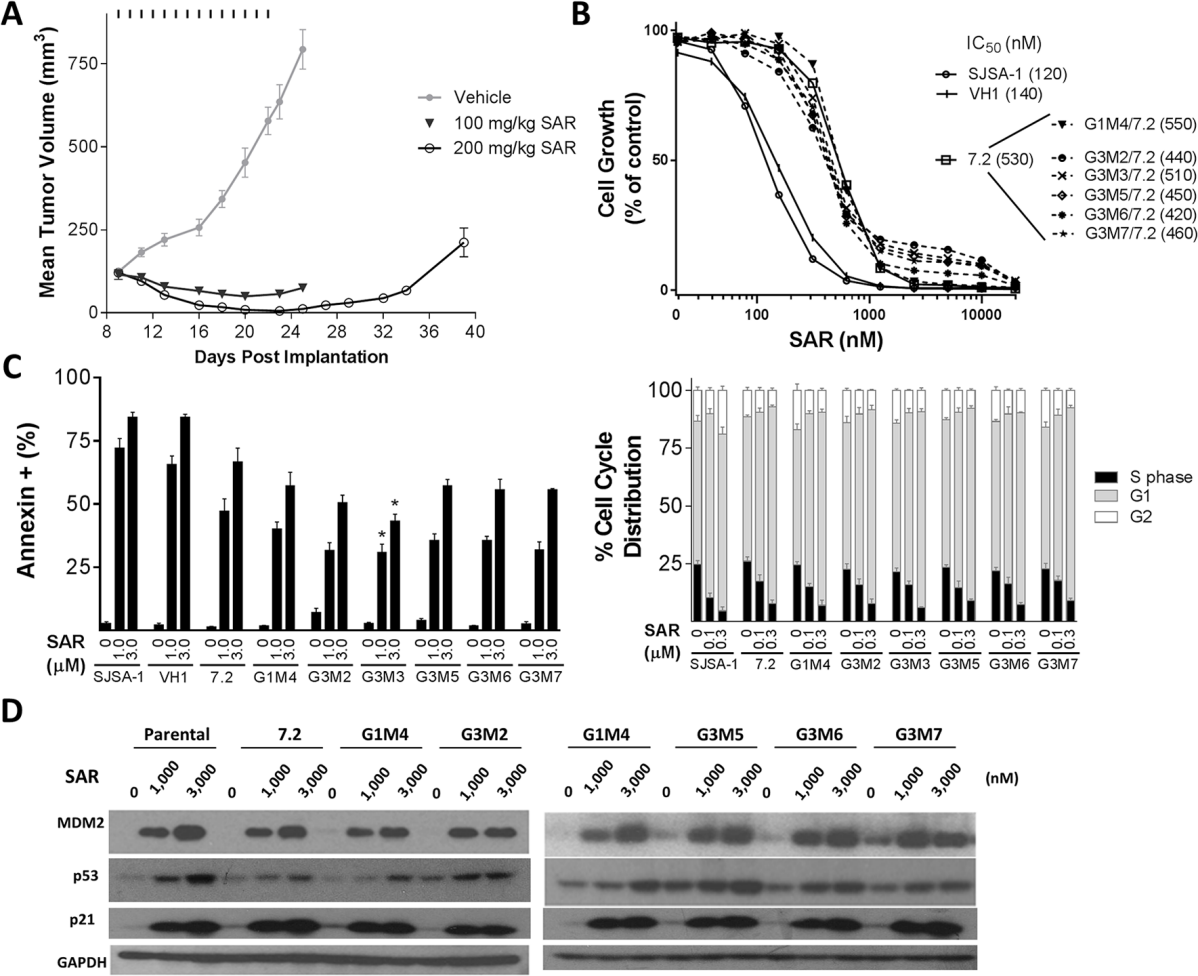
Establishment and characterization of 7.2 sublines from treatment of 7.2 SJSA-1xenograft tumors with SAR405838 in mice. A, Xenograft tumors established from the 7.2 SJSA-1 subline were treated with vehicle, 100 mg/kg or 200 mg/kg of SAR405838 orally for 14 days. When tumors treated with 200 mg/kg of SAR405838 grew to 200 mm^3^, they were harvested and cultured to establish several 7.2 sublines (G3M2, G3M3, G3M5-G3M7). One subline (G1M4/7.2) was also established by harvesting and culturing one 7.2 xenograft tumor treated with vehicle control. B, Cell growth inhibition of SAR405838 in G3M2, G3M3, G3M5-G3M7 sublines. VH1 and SJSA-1 parental cell lines were included as control cell lines. Cells were treated with SAR405838 for 4 days and cell viability was evaluated by a WST assay. C, Analysis of apoptosis and cell cycle in G3M2, G3M3, G3M5-G3M7 sublines, 7.2 subline, SJSA-1 parental cell line and VH1 cell line. Cells were treated with SAR405838 for 48 h for apoptosis analysis by flow cytometry with Annexin V/P.I. double staining. The test was used to compare sublines (G2M1, G2M2, G2M4-G2M8) to 7.6 subline. *, *P* < 0.05. Cells were treated with SAR405838 for 24 h for cell cycle analysis by flow cytometry with P.I. staining. D, Western blot analysis of p53, MDM2, p21 and GAPDH protein level in SJSA-1 parental, 7.2 subline, G1M4/7.2, G3M2, G3M5, G3M6, and G3M7 sublines. Cells were treated for 24 hr with SAR405838 at indicated concentrations.

To this end, xenograft tumors were developed in mice using the SJSA-1 7.2 subline and treated with 100 or 200 mg/kg of SAR405838, daily for 14 days (Fig 4A). While SAR405838 at 100 mg/kg induced tumor regression by 50% on average, it failed to achieve complete regression on any tumor (Fig 4A). In comparison, SAR405838 at 200 mg/kg achieved complete tumor regression with no evidence of toxicity in 5 out of 8 mice by the end of the treatment (Fig 4A and S1D Fig), but all regressed tumors regrew after the termination of the treatment. When those 5 regressed tumors regrew to an average volume of 200 mm^3^, they were harvested and cultured to establish 5 sublines (G3M2, G3M3, G3M5-G3M7). In a cell growth assay, these five 7.2 sublines are 4–5 times less sensitive to SAR405838 than the SJSA-1 parental cell line (Fig 4B). Moreover, these five 7.2 sublines, a subline established from a 7.2 xenograft tumor treated with vehicle (G1M4) and the original 7.2 subline are all equally sensitive to SAR405838 (Fig 4B). Flow cytometric analysis showed that SAR405838 is effective in induction of apoptosis in these five 7.2 sublines (G3M2, G3M3, G3M5- G3M7), with potencies modestly less than that observed in the parental SJSA-1 cell line (Fig 4C). Additionally, the flow cytometric analysis also showed SAR405838 to be equally potent in induction of cell cycle arrest in these five 7.2 sublines, the vehicle-treated 7.2 subline (G1M4) and the SJSA-1 parental cell line (Fig 4C).

We next analyzed the p53 mutation status of these five SJSA-1 7.2 sublines (G3M2, G3M3, and G3M5-G3M7) and found that all 5 sublines still have a single heterozygous C176F p53 mutation, but no additional p53 mutation (S4 Table). To assess if p53 is still functional in these sublines, we examined induction of p21 and MDM2 and accumulation of p53 protein by SAR405838. In the four representative sublines, SAR405838 effectively induces a dose-dependent increase of MDM2, p21 and p53 proteins (Fig 4D), indicating that p53 is still functional in these sublines.

Taken together, these data show that when tumors established from the 7.2 subline with a single heterozygous C176F p53 mutation were further treated with SAR405838 at a higher dose, several tumors underwent regression. Five sublines established from the regrown tumors contain the single heterozygous C176F p53 mutation from the original 7.2 subline, but have no additional p53 mutation. Consistently, p53 is still functional in these sublines, which are equally sensitive to SAR405838, when compared to the original 7.2 subline.

### Comparison of SJSA-1 sublines obtained from *in vitro* and *in vivo* treatment with SAR405838

We next directly compared the parental SJSA-1 and several representative sublines, obtained from either *in vitro* or *in vivo* SAR405838 treatment, in several assays to assess their sensitivity to SAR405838. For these comparisons, we selected the MIR2 subline obtained from *in vitro* treatment, the 7.2 subline containing a single heterozygous C176F p53 mutation established from a regressed and regrown SJSA-1 tumor treated with one round of SAR405838, the 7.6 subline established from a regressed and regrown SJSA-1 tumor treated with one round of SAR405838 but containing no p53 mutation, the G2M6 subline containing a single heterozygous C176F p53 mutation obtained from a 7.6 tumor with prolonged SAR405838 treatment and the G3M6 subline containing a single heterozygous C176F p53 mutation obtained from a 7.2 tumor treated with additional SAR405838.

**Fig 5:**
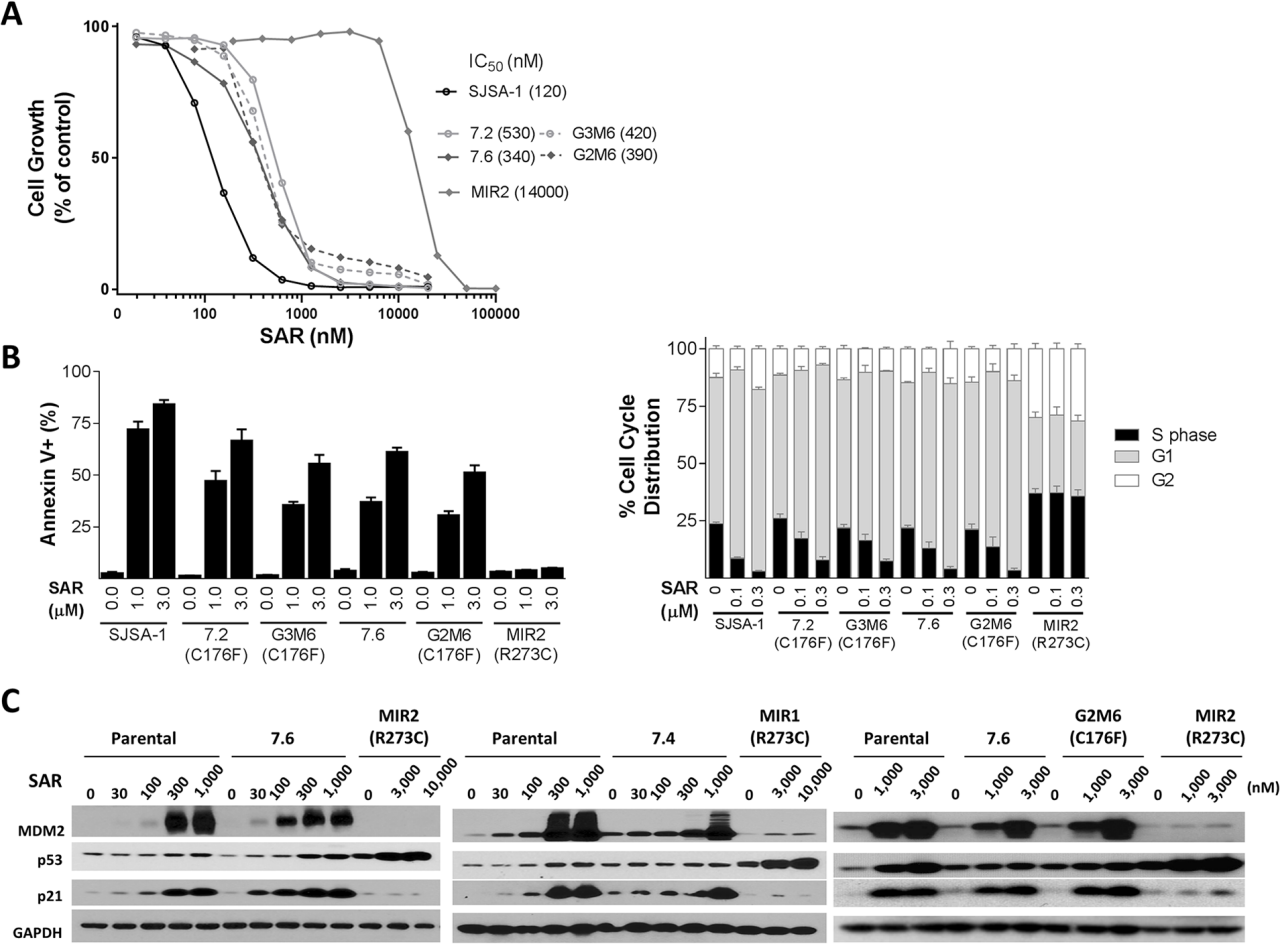
Direct comparison of the activity of SAR405838 in SJSA-1 parental and representative sublines obtained from either *in vitro* or *in vivo* treatment with SAR405838. A, Cell growth inhibition of SAR405838 in SJSA-1 parental and representative *in vitro* and *in vivo* sublines. Cells were treated with SAR405838 for 4 days and cell viability was evaluated by a WST assay. B, Analysis of apoptosis and cell cycle in SJSA-1 parental and representative *in vitro* and *in vivo* sublines. Cells were treated with SAR405838 for 48 h for apoptosis analysis by flow cytometry with Annexin V/P.I. double staining. The test was used to compare sublines (G2M1, G2M2, G2M4-G2M8) to 7.6 subline. *, *P* < 0.05. Cells were treated with SAR405838 for 24 h for cell cycle analysis by flow cytometry with P.I. staining. C, Western blot analysis of p53, MDM2, p21 and GAPDH protein level in SJSA-1 parental, and representative *in vitro* and *in vivo* sublines. Cells were treated for 24 hr with SAR405838 at indicated concentrations.

In a cell growth inhibition assay, the MIR2 subline is 100-times less sensitive to SAR405838 than the parental SJSA-1 cell line based upon the IC_50_ values (Fig 5A). Interestingly, all the four representative sublines obtained from *in vivo* tumor treatment with SAR405838 have the same sensitivity to the drug in the cell growth assay (Fig 5A), regardless of whether they retain p53 wild-type or a single heterozygous C176F p53 mutation or whether they are obtained with either one round or multiple rounds of the drug treatment in mice. These *in vivo*-obtained sublines are approximately 3–5 times less sensitive to SAR405838 than the parental SJSA-1 cell line, but are >20-times more sensitive to the drug than the MIR2 subline obtained from *in vitro* treatment with the drug (Fig 5A).

Flow cytometric analysis showed that although SAR405838 is ineffective in inducing apoptosis in the MIR2 subline, it effectively induces apoptosis in a dose-dependent manner in the parental SJSA-1 and all sublines obtained *in vivo* (Fig 5B). The potencies of SAR405838 in induction of apoptosis in the 7.2, 7.6, G2M6 and G3M6 sublines are moderately less than in the parental SJSA-1 cell line (Fig 5B). Furthermore, while SAR405838 effectively and dose-dependently induces cell cycle arrest in the parental SJSA-1 and all sublines obtained from *in vivo* treatment, it has no effect in the MIR2 subline obtained from *in vitro* treatment (Fig 5B).

Western blot analysis showed that while SAR405838 effectively and dose-dependently induces upregulation of p53, MDM2 and p21 proteins in the parental SJSA-1 and all the sublines obtained from *in vivo* SAR405838 treatment, it fails to induce robust upregulation of p53, p21 and MDM2 in both the MIR1 and MIR2 sublines obtained from *in vitro* SAR405838 treatment (Fig 5C).

These data clearly show that there is a marked difference in the response to SAR405838 between those SJSA-1 sublines obtained from *in vitro* SAR405838 treatment and those sublines obtained from *in vivo* SAR405838 treatment.

### Computational modeling of the interaction between the C176F p53 mutation and DNA

Our data clearly show that SAR405383 can effectively induce p53 activation in all SJSA-1 sublines containing a single heterozygous C176F p53 mutation. In the crystal structure of wild-type p53 in a complex with DNA (PDBID: 4HEJ) [32], C176, together with H179, C238, C242 residues, coordinates with a zinc ion and appears to be very critical in maintaining the structural integrity of p53 [40,41]. Therefore, it was surprising that p53 with a heterozygous C176F mutation is functionally active as a transcriptional factor. Hence, we performed computational simulations to shed light on the structure for p53 with a heterozygous C176F p53 mutation.

**Fig 6:**
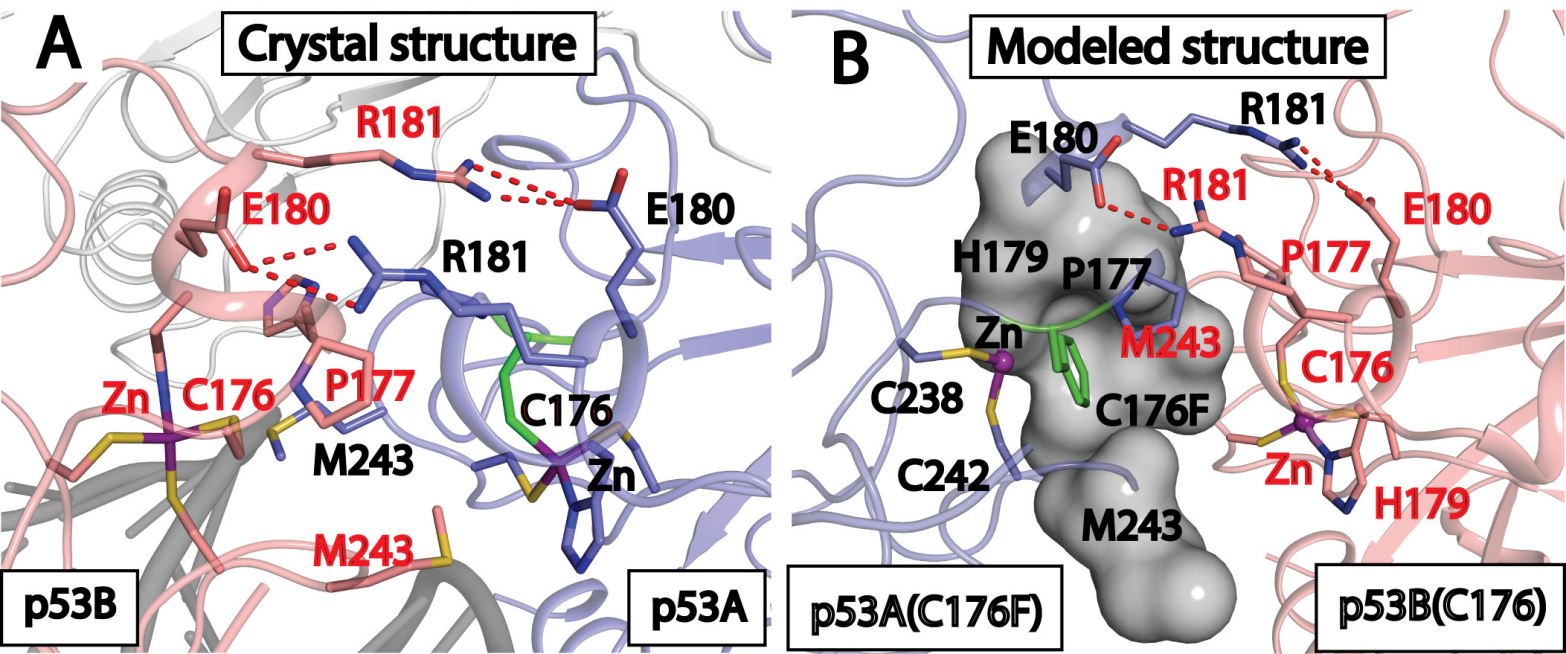
Computational modeling of p53 tetrameric structure containing a single heterozygous C176F p53 mutation in complex with DNA. A, The loop-sheet-helix interaction interface between two p53 molecules revealed by the crystal structure (PDB entry: 4HJE). The backbone of DNA is shown in dark grey, the zinc ion is purple and hydrogen bonds are depicted as red dashed lines. B, The additional interactions gained in p53 dimer contacting a wild-type (colored in pink) and a C176F mutant (colored in blue) p53 at the loop-sheet-helix interaction interface based on the final snapshot of conformation from the 6 ns MD simulation of the FCCF model with DNA (not shown). The interactions include hydrophobic interaction between C176F and two M243 residues and the cation-aromatic interaction between C176F and the zinc ion.

For gene transcription, p53 function as a tetramer in which two p53 monomers form a dimer to interact with DNA and two p53 dimers interact loosely to form a functional tetramer. For wild-type p53, two p53 monomers interact with each other *via* hydrophobic interactions between P177 and M243 residues and the salt-bridge interaction between R181 and E180 residues from respective monomers (denoted as p53A and p53B, respectively), as shown in Fig 6A [41]. In a 6 ns molecular dynamics (MD) simulation of the wild-type p53 tetramer complexed with DNA, we found these interactions are well-maintained, as compared to those in the crystal structure[32] (S5 Table).

In cell lines containing a C176F heterozygous p53 mutation, each p53 tetramer consists of two p53 wild-type molecules and two C176F p53 mutants and consequently, there are a total of six possible structural models for a p53 tetramer. In four of these six models, a wild-type p53 monomer molecule and a C176F p53 mutant monomer form a dimer which interacts with DNA. In the two other models, two p53 wild-type monomers form a dimer and two C176F p53 mutant monomers form another dimer to interact with DNA. We denote these arrangements as FCCF, FCFC, CFCF, CFFC, FFCC, and CCFF, with C representing the wild-type p53 (C176) monomer and F the C176F mutated p53 monomer (S5 Table and S2 Fig). We used the motional fluctuations of the p53 backbone atoms in these models from the p53 wild-type tetramer crystal structure to assess the structural stability for these 6 models containing a C176F heterozygous mutation. Our data showed that the FFCC model appears to be least stable and the FCCF tetramer appears to be the most stable. In each of the dimers, we also calculated the salt-bridge interaction distances between R181 and E180 as another indicator of the structural stability of the dimer. The most stable FCCF tetramer also has a stronger R181—E180 interaction than the other five models but this interaction is slightly weaker than that in the wild-type p53 tetramer (S2 Fig).

Since the mutation of C176F results in a loss of zinc coordination, we analyzed the model structures of the FCCF tetramer from the simulations and compared them to those in the wild-type p53 tetramer to identify additional stabilizing interactions. We found that the mutated Phe residue gains additional hydrophobic interactions with the M243 residue in the same monomer, as well as the M243 residue from the other monomer (Fig 6B). Moreover the mutated Phe residue also engages in cation-aromatic interactions with the zinc ion (Fig 6B). Both interactions contribute to stabilization of the loop-sheet-helix motif structure between the mutant and the wild-type p53 dimer. Hence, our modeling suggests that the p53 FCCF tetramer has gained additional interactions through the mutated Phe residues and these additional interactions compensate, at least partially for the loss of the zinc coordination with the C176 residues in the wild-type p53 tetramer structure.

## Discussion

The SJSA-1 osteosarcoma cell line, containing wild-type p53 and an amplified MDM2 gene, is sensitive to MDM2 inhibitors and has been used extensively as a model with which to investigate the efficacy and mechanism of action for different classes of MDM2 inhibitors. In our previous study[19], we showed that SAR405838, a potent MDM2 inhibitor currently in clinical development, is highly effective in inhibition of cell growth and induction of apoptosis *in vitro* in the SJSA-1 cell line and induces rapid and complete tumor regression against SJSA-1 xenograft tumors in mice. In the present study, we have employed the SJSA-1 cell line to investigate the acquired resistance mechanisms for SAR405838 *in vitro* and *in vivo*. The current study has revealed some marked differences in the development of acquired drug resistance of SJSA-1 cells treated with a potent, specific and highly efficacious MDM2 inhibitor *in vitro* and *in vivo*.

Our data show that treatment of the SJSA-1 cells *in vitro* with either a fixed concentration of SAR405838 or with stepwise increments of concentration of SAR405838 yields highly resistant subline lines (CMIR, MIR1 and MIR2), which are 100-times less sensitive to the drug than the parental SJSA-1 cell line based upon IC_50_ values. These resistant cell lines do not undergo apoptosis and cell cycle arrest when treated with SAR405838 *in vitro*. Furthermore, SAR405838 is completely ineffective against the MIR2 xenograft tumors in mice, in contrast to the rapid and complete tumor regression achieved by SAR405838 in the xenograft tumors established *in vitro* using the SJSA-1 parental cell line. SAR405838 is unable to induce p53 activation in these three *in vitro* resistant cell lines, and this stands in contrast to the dose-dependent and robust activation of p53 by SAR405838 in the SJSA-1 parental cell line.

Since the activity of MDM2 inhibitors depends upon wild-type p53, we sequenced p53 in these resistant cell lines and found that each of them contains a heterozygous, but not a homozygous p53 mutation. While both the MIR1 and MIR2 sublines contain a single R273C p53 heterozygous mutation, the CMIR subline contains two heterozygous p53 mutations, L130R and C277F. The "hot spot" R273C mutation identified in both the MIR1 and MIR2 sublines and the C277F mutation identified in the CMIR subline are common DNA contact mutations [41]. While R273 plays a crucial role in docking p53 to the DNA backbone, C277 makes direct contacts with DNA bases [42] and mutations at these positions dramatically reduce the DNA binding affinity [43,44]. Our data also show that different inactivating mutations of p53 can occur when the same cell line is treated with an MDM2 inhibitor *in vitro* using two different treatment protocols. These *in vitro* data are consistent with a previous study [23] with the SJSA-1 cell line and nutlin-3, which showed that treatment *in vitro* of the SJSA-1 cell line with nutlin-3 generates several highly resistant sublines harboring inactivating, “hot-spot” p53 mutations.

In contrast to the profound resistance obtained by treatment of the SJSA-1 parental cell line *in vitro* with SAR405838 (Fig 1), treatment with SAR405838 of the SJSA-1 xenograft tumors in mice fails to generate highly resistant tumor sublines to the drug. When xenograft tumors established using the SJSA-1 parental cell line were treated with SAR405838 at 100 mg/kg, daily for two weeks, all of the tumors underwent rapid and complete regression (Fig 2A), similar to what we have observed in our previous study [19]. Furthermore, we have shown that SAR405838 is highly effective in induction of strong and persistent apoptosis in the SJSA-1 tumor tissue with a single, oral dose at 100 mg/kg[19]. Despite the strong apoptosis induction by SAR405838 in the SJSA-1 tumor tissue, all regressed tumors eventually returned (Fig 2A). Harvesting and culturing regrown tumors established 5 sublines (sublines 7.2–7.4, 7.6, and 7.7). Evaluation of these 5 sublines, together with one subline established from a vehicle-treated tumor and the parental cell line, demonstrated that these 5 sublines are still responsive to SAR405838 in assays of cell growth inhibition, induction of apoptosis and cell cycle arrest, albeit with 3–5 times reduced sensitivity as compared to the vehicle-treated subline or the parental cell line (Fig 2). Consistently, SAR405838 effectively and dose-dependently activates p53 in these 5 sublines, also with reduced potency as compared to that in the parental cell line. Interestingly, p53 sequencing showed that while the 7.2 subline contains a heterozygous C176F p53 mutation, the other four sublines have no p53 mutation detected.

To assess if longer treatment of the SJSA-1 tumors in mice with SAR405838 can generate highly resistant sublines, we treated xenograft tumors established in mice using the 7.6 subline with SAR405838 daily at 100 mg/kg for 14 days (Fig 3). At the end of this treatment SAR405838 was found to induce partial regression of every single tumor by 72% on average but did not yield complete regression of any tumor, and the tumors resumed their growth after the treatment was stopped. Treatment of the regrown tumors with 200 mg/kg of SAR405838 daily for 8 days, effectively halted tumor growth but did not lead to regression; the tumors resumed growth again once the treatment was ended (Fig 3). These data showed that while tumors established from the 7.6 subline are responsive to SAR405838, they are less sensitive to SAR405838 than tumors established using the SJSA-1 parental cell line. To determine if the additional treatments (14 days at 100 mg/kg, followed by 8 days at 200 mg/kg) generated highly resistant sublines, we harvested and cultured the SAR405838-treated tumors when their size reached 500 mm^3^, and established 7 sublines. In assays of cell growth, apoptosis and cell cycle progression, these 7 sublines are equally sensitive to SAR405838 as compared to the 7.6 subline and are only 3–5 times less sensitive than the parental SJSA-1 cell line. In these sublines SAR405838 was consistently able to induce dose-dependent and robust activation of p53 based upon upregulation of p53, p21 and MDM2 proteins. Sequencing of p53 showed that each of these 7 sublines now harbors a heterozygous C176F p53 mutation, the same as that identified in the 7.2 subline.

The 7.2 subline contains a heterozygous C176F p53 mutation but is only modestly less sensitive to SAR405838 than the parental cell line in assays of cell growth inhibition, apoptosis and cell cycle progression. We tested if more profound resistance could be developed if xenograft tumors established using the 7.2 subline were further treated with SAR405838 in mice (Fig 4). While SAR405838 at 100 mg/kg daily for 14 days induced regression of the same tumors by 50% on average, it failed to induce complete tumor regression on any tumor. In comparison, SAR405838 at 200 mg/kg daily for 14 days was able to achieve complete tumor regression in five out of eight tumors and more than 85% tumor regression in the remaining three tumors. These data showed that the xenograft tumors established using the 7.2 subline are still responsive to SAR405838, albeit with somewhat reduced sensitivity as compared to the xenograft tumors established using the parental SJSA-1 cell line. When those five completely regressed tumors regrew, we established 5 new sublines. Evaluation of these five sublines *in vitro* showed that SAR405838 is very effective in inhibition of cell growth and induction of apoptosis and cell cycle arrest in each of them, with a potency 3–5 times less than that in the parental SJSA-1 cell line and SAR405838 effectively induces dose-dependent p53 activation in these sublines. p53 sequencing showed that all the five sublines contain the same single heterozygous C176F p53 mutation, but no additional p53 mutation. Taken together, our investigation using both the 7.6 and 7.2 sublines demonstrates that prolonged treatment of the SJSA-1 tumors with SAR405838 *in vivo* fails to yield highly resistant tumor cell lines. Although a single heterozygous C176F p53 mutation is detected in many of the sublines established after tumor regression in the treatment with SAR405838, p53 can be still effectively activated by SAR405838 in these sublines.

Based upon the crystal structure of the p53:DNA complex,[32] the C176 residue of p53, together with H179, C238, and C242 residues, coordinates with a zinc ion. Hence, mutation of the C176 residue to phenylalanine in p53 eliminates the coordination of C176 with the zinc and could have a major negative impact on the structural stability of the p53 protein. Therefore, it was surprising to find that SAR405838 can effectively activate p53 in all sublines containing a single heterozygous C176F p53 mutation. We performed computational modeling to assess the impact of the single heterozygous C176F p53 mutation on the p53: DNA complex structure. Since each p53 functional unit is a tetramer with four p53 monomer components, a total of 6 structural tetramer models with 2 mutated p53 and 2 wild-type p53 molecules are possible and were investigated. Among the six possible structural models, the FCCF model was found to be the most stable. Our modeling further showed that in the FCCF tetramer model, the two mutated Phe residues gain additional hydrophobic and cation-π interactions, compared to the wild-type p53 tetramer structure, which help to maintain the structural stability and transcriptional activity of the p53 tetramer containing a heterogamous C176F mutation.

The profound resistance developed by treating the SJSA-1 cells *in vitro* with SAR405838 and the modest resistance developed by treating the SJSA-1 xenograft tumors in mice with SAR405838 indicate that even using the same tumor cell line and the same drug, tumor cells *in vitro* or *in vivo* may have a different response in the development of resistance to drug treatment. When the SJSA-1 tumor cells are treated with SAR405838 *in vitro*, the cells are under constant high pressure from the drug and p53 is continually activated. In order for the tumor cells to survive, there is a need to acquire an inactivating p53 mutation to render the drug completely ineffective. On the other hand, when the SJSA-1 tumors are treated with SAR405838 in mice, although the drug reaches sufficient exposure and duration to induce strong p53 transcriptional activation and robust apoptosis in the tumor tissue, p53 activation is not constant and fluctuates as a result of drug pharmacokinetics [19]. Tumor cells do not, therefore, need to acquire inactivating p53 mutation(s) in order to survive. Instead, a structural mutation (C176F) of p53, which causes tumor cells to become 3–5 times less sensitive to the drug *in vitro*, appears to be sufficient for some tumor cells to survive and for tumors to regrow after the treatment is stopped.

The results of this study have an implication for investigation of acquired drug resistance mechanisms for other classes of anticancer drugs. In investigation of drug resistant mechanisms, it has been assumed that the findings obtained from *in vitro* cell culture models may help to understand the drug resistant mechanisms in patients. Although it may be true in some cases, our present study suggests that there may be very different acquired resistant mechanisms for the same drug between *in vitro* and *in vivo* settings.

## Supporting Information

S1 Fig Body weight change of mice in Fig 1D (A), Fig 2A (B), Fig 3A (C) and Fig 4A (D). Data shown are mean ± SEM for 6–8 mice.
(TIF)

S2 Fig The root-mean-square deviations of the p53 backbone atoms from the wild-type crystal structures in four different tetrameric arrangements from the 6 ns MD simulations. The distances between R181 (Cζ) of E180 (Cδ) in the pair of demerit p53 proteins calculated from the MD simulations. The standard deviations were shown in error bars. The arrangement of monomer p53 denoted as A, B, C and D was shown in the legend.
(TIF)

S1 Table p53 Sequencing of In Vivo Acquired Resistant Sublines. A, p53 sequencing of parental SJSA-1, vehicle treated SJSA-1 sublines and *in vitro* resistant SJSA-1 sublines. B, p53 sequencing of *in vitro* resistant MIR2 sublines established after *in vivo* treatment with vehicle (G1M3) or 200 mg/kg/day of SAR405838 for two weeks (G3M1, G3M3 and G3M6).
(TIF)

S2 Table p53 sequencing of SJSA-1 sublines established after *in vivo* treatment with 100 mg/kg/day of SAR405838 for two weeks. (TIF)

S3 Table p53 sequencing of 7.6/SJSA-1 sublines (no mutation) established after three rounds of *in vivo* SAR405838 treatment. (TIF)

S4 Table p53 sequencing of 7.2/SJSA-1 subline (mutation C176F) established after *in vivo* treatment with 200 mg/kg/day of SAR405838 orally for two weeks. (TIF)

S5 Table Root-mean square deviations of the wild-type and mutant p53 backbone atoms from the crystal structure and the distances between R181(Cz) and E180(Cd) in each dimeric p53 protein determined from the MD simulations. (TIF)
