# Supplementary figures and images for: Significant Differences in the Development of Acquired Resistance to the MDM2 Inhibitor SAR405838 between In Vitro and In Vivo Drug Treatment
Source: PLoS One. 2015 Jun 12;10(6):e0128807. doi: 10.1371/journal.pone.0128807 (PMC4466389; doi:10.1371/journal.pone.0128807)

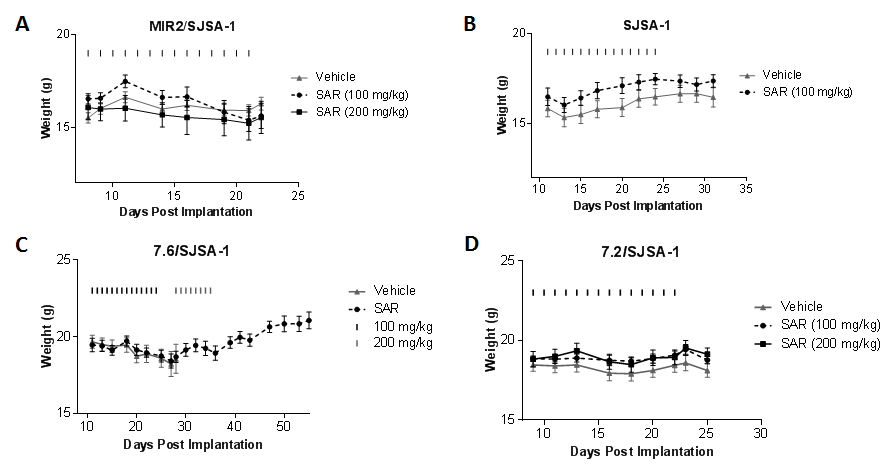

Supplement: S1 Fig — Data shown are mean ± SEM for 6–8 mice. (TIF) [file pone.0128807.s001.tif]

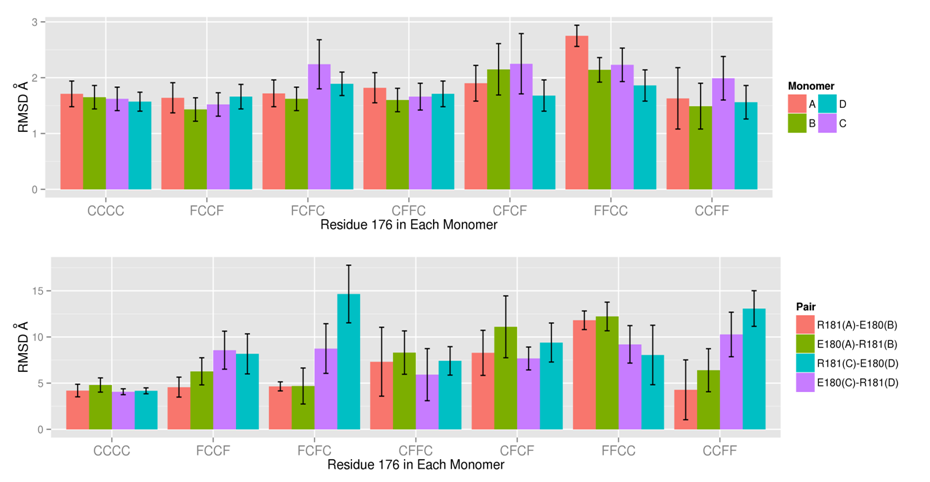

Supplement: S2 Fig — The distances between R181 (Cζ) of E180 (Cδ) in the pair of demerit p53 proteins calculated from the MD simulations. The standard deviations were shown in error bars. The arrangement of monomer p53 denoted as A, B, C and D was shown in the legend. (TIF) [file pone.0128807.s002.tif]

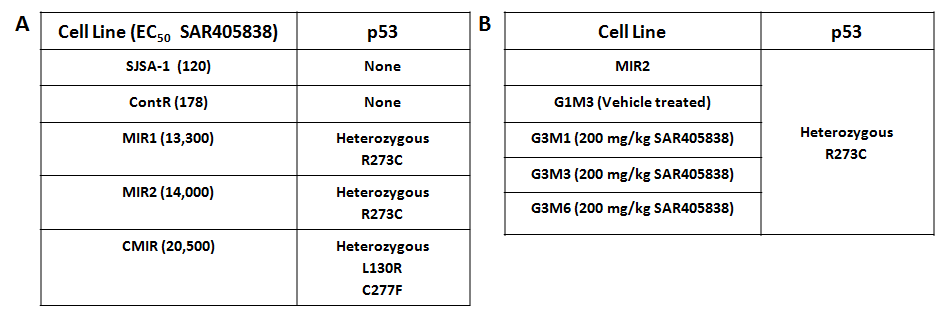

Supplement: S1 Table — A, p53 sequencing of parental SJSA-1, vehicle treated SJSA-1 sublines and in vitro resistant SJSA-1 sublines. B, p53 sequencing of in vitro resistant MIR2 sublines established after in vivo treatment with vehicle (G1M3) or 200 mg/kg/day of SAR405838 for two weeks (G3M1, G3M3 and G3M6). (TIF) [file pone.0128807.s003.tif]

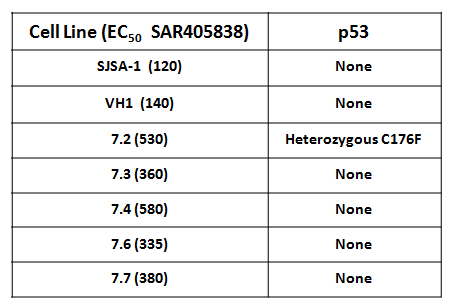

Supplement: S2 Table — (TIF) [file pone.0128807.s004.tif]

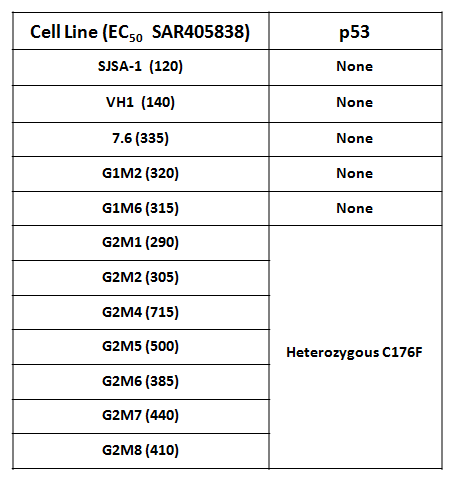

Supplement: S3 Table — (TIF) [file pone.0128807.s005.tif]

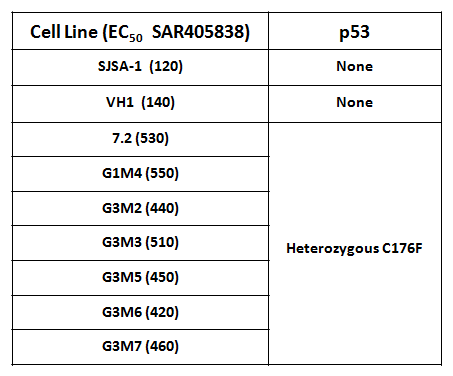

Supplement: S4 Table — (TIF) [file pone.0128807.s006.tif]

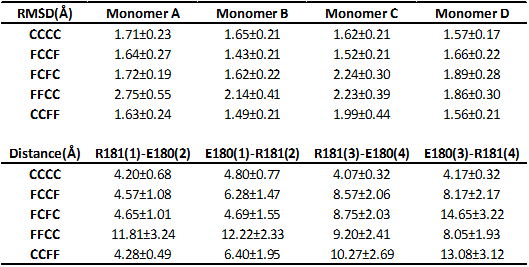

Supplement: S5 Table — (TIF) [file pone.0128807.s007.tif]
